# Supplementary material for: Splice-Junction-Based Mapping of Alternative Isoforms in the Human Proteome
Source: Cell Rep. Author manuscript; Available in PMC 2020 Jan 15. (PMC6961840; doi:10.1016/j.celrep.2019.11.026)
Supplement: 2 [file NIHMS1546469-supplement-2.zip › DF1/PXD009737/TestisTrypsin/TestisTrypsin_63_TMEM120A_HLPVGSHADVGDCLRDWEDLQQDFQNIQETHR.pdf]

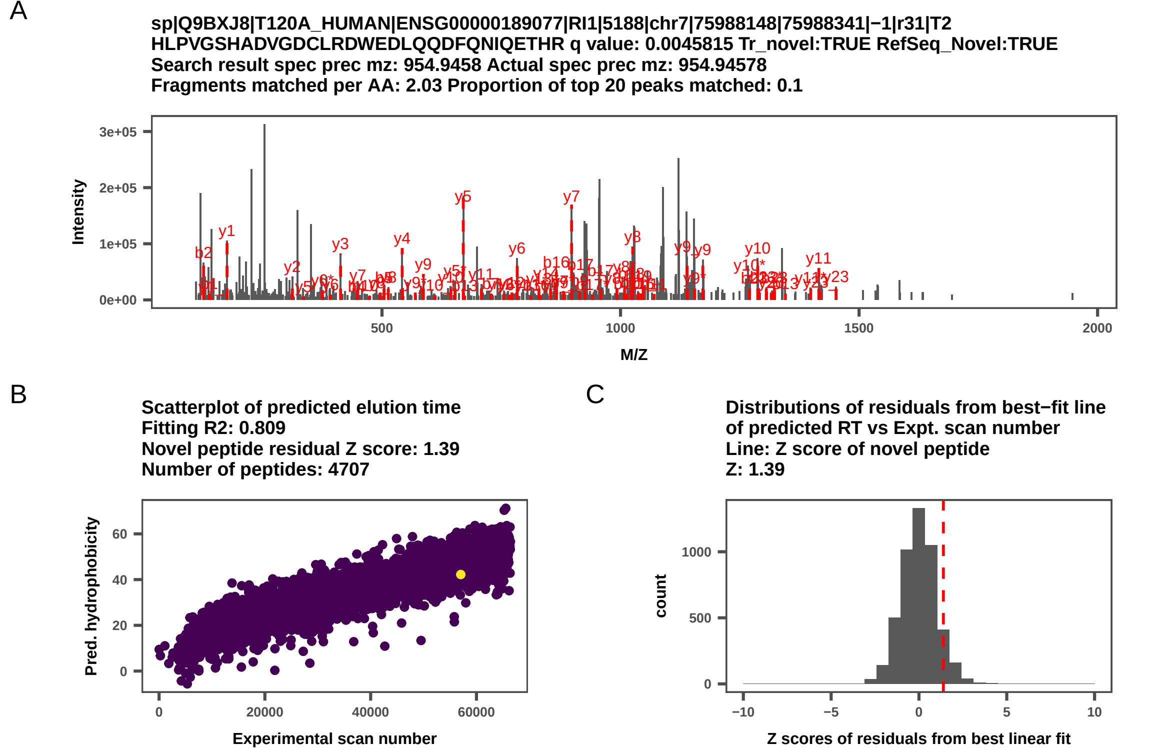
A
sp|Q9BXJ8|T120A_HUMAN|ENSG00000189077|RI1|5188|chr7|75988148|75988341|−1|r31|T2
HLPVGSHADVGDCLRDWEDLQQDFQNIQETHR q value: 0.0045815 Tr_novel:TRUE RefSeq_Novel:TRUE
Search result spec prec mz: 954.9458 Actual spec prec mz: 954.94578
Fragments matched per AA: 2.03 Proportion of top 20 peaks matched: 0.1
Intensity
3e+05
2e+05
1e+05
0e+00
M/Z
500
1000
1500
2000
b2
y1
y2
y3
y4
y5
y6
y7
y8
y9
y10
y10*
y11
B
Scatterplot of predicted elution time
Fitting R2: 0.809
Novel peptide residual Z score: 1.39
Number of peptides: 4707
Pred. hydrophobicity
60
40
20
0
0
20000
40000
60000
Experimental scan number
C
Distributions of residuals from best−fit line
of predicted RT vs Expt. scan number
Line: Z score of novel peptide
Z: 1.39
count
1000
500
0
−10
−5
0
5
10
Z scores of residuals from best linear fit
